# Comparison of in vitro properties of periodontal ligament stem cells derived from permanent and deciduous teeth

**DOI:** 10.15171/joddd.2017.026

**Published:** 2017-09-20

**Authors:** Masoumeh Khoshhal, Iraj Amiri, Leila Gholami

**Affiliations:** ^1^Dental Implant Research Center, Hamadan University of Medical Sciences, Hamadan, Iran; ^2^Department of Anatomy and Embryology, Faculty of Medicine, Hamadan University of Medical Sciences, Hamadan, Iran; ^3^Department of Periodontology, Faculty of Dentistry, Hamadan University of Medical Sciences, Hamadan, Iran

**Keywords:** Deciduous tooth, mesenchymal stem cells, periodontal ligament, permanent dentition

## Abstract

***Background.*** Stem cells have contributed to the development of tissue-engineered-based regenerative periodontal therapies. In order to find the best stem cell sources for such therapies, the biologic properties of stem cells isolated from periodontal ligaments (PDL) of deciduous (DePDLSC) and permanent (PePDLSC) teeth were comparatively evaluated.

***
Methods.*** PDL stem cells were isolated from six sound fully erupted premolars and six deciduous canines of healthy subjects. In vitro biologic characteristics such as colony formation, viability, stem cell marker identification and osteogenic differentiation (using alkaline phosphatase analysis and Alizarin red staining) were comparatively assessed using one-way ANOVA and post hoc Tukey tests using SPSS 13.0.

***
Results.*** Stem cell populations isolated from both groups were CD105+ and CD90+ and CD45‒. No statistically significant differences were found in stem cell markers, colony formation and viability. Both groups were capable of osteogenic differentiation. However, alkaline phosphatase activity test showed a statistically significant difference, with PePDLSC exhibiting higher alkaline phosphatase activity (P=0.000). No statistically significant difference was seen in quantitative alizarine red staining (P=0.559).

***
Conclusion.*** Mesenchymal stem cells of PDL could successfully be isolated from permanent and deciduous teeth. A minor difference was observed in the osteogenic properties of the two cell types, which might affect their future clinical applications.

## Introduction

‏In the fascinating field of stem cell biology and tissue engineering successful regeneration of lost periodontal tissues is still an important and growing area of research in periodontology. Formation of a new connective tissue attachment to the root surface is the fundamental goal in regeneration of the periodontium. This requires the simultaneous regeneration of cementum, the periodontal ligament and the alveolar bone.^1,2^ Since PDL tissue and its cells are a key factor in the periodontal regeneration process recent studies have focused on tissue engineering and stem cell therapies using cells derived from the PDL.

Identification and characterization of suitable tooth-derived stem cell populations have been evaluated in tissue engineering studies in dentistry. Many parts of teeth have been used, leading to successful isolation of stem cells. To date 5 different human dental stem/progenitor cells have been isolated and characterized. ‘Postnatal dental pulp stem cells’ (DPSCs) were the first stem cells isolated from tooth structures.^3^ Subsequently, three more types of dental-MSC-like populations were isolated and characterized: stem cells from exfoliated deciduous teeth (SHED),^4^ periodontal ligament stem cells (PDLSCs)^5^ and stem cells from the apical papilla (SCAP).^6,7^ A more recent stem cell population, referred to as ‘dental follicle precursor cells’ (DFPCs), has also been successfully isolated.^8^

The concept that stem cells may reside in the periodontal tissues was proposed approximately 20 years ago by Melcher.^9^ Studies by McCulloch et al provided the primary evidence of stem cells residing within the periodontal tissues adjacent to blood vessels within the periodontal ligament.^10^

After isolation of PDLSCs from normal impacted third molars by Seo et al in 2004, using cloning techniques they verified that only some of the progenitor cell strains of periodontal ligament can be considered stem cells.^5^ These periodontal adult stem cells express a variety of stromal cell markers and have the morphological, phenotypic and proliferative characteristics of adult MSCs.^11^ They can promote tissue turnover and homeostasis, and serve as a source of renewable progenitor cells, generating cementoblasts, osteoblasts and fibroblasts throughout the adult life. They have also shown tissue regeneration capacity and osteogenic cementogenic and PDL-like tissue differentiation and are considered as the most promising source of stem cells for periodontal regenerative therapies.^6,12-18^

PDLSCs have also been isolated from the remains of PDL tissue on the alveolar bone surface of extraction sockets. These cells have shown a higher osteogenic/adipogenic differentiation potential than those from the PDL of the root surface.^12,19^ More recent investigations have been able to isolate PDLSCs from inflamed PDL tissue and these cells have been shown to retain the potential to regenerate cementum and related PDL tissues.^20^

Interestingly it seems that stem cell origin is an important factor affecting their properties and differentiation capacities. For instance, PDL-derived stem cells have shown higher expression of type I collagen compared to stem cells isolated from the pulp.^21,22^

Donor age also seems to be a factor affecting stem cell properties and their regenerative capacities.^23^ Recent findings have shown a loss of the proliferation and differentiation potential of human PDLSCs with advancing age of donors. ^24^

There is still ongoing search for finding an intraoral source of stem cells for periodontal regeneration with ideal properties.^19,25-27^

A few resent studies have been able to identify PDLSC in the PDLs of deciduous teeth, suggesting a new and younger tissue source for PDLSC isolation.^28,29,30^ Deciduous teeth stem cells have recently attracted more attention due to their simple isolation and lack of ethical controversy, and negligible immunogenicity in some studies^31-33^ However, reports have been controversial and the best type of PDL stem cell type is still not certainly determained.^34^

In addition, the irretrievability of PDLSC after cryopreservation makes these kinds of cells possible candidates for stem cell banking and future application in periodontal tissue engineering.^35,36^

To determine whether deciduous stem cells are suitable candidates for future applications in regenerative therapies, this study, we comparatively assessed the in vitro characteristics of mesenchymal progenitor cells (MPC) harvested from the PDL of deciduous and permanent teeth.

## Methods

Six fully erupted premolars from 4 healthy subjects (one male and three females, 16‒20 years of age) and six deciduous canines of 3 healthy subjects (two males and two females, 9‒10 years of age), with no signs of root resorption or ankylosis, which were to be extracted for orthodontic treatment, were collected after the patients or their parents signed a consent form. The study protocol, which was in accordance with the Helsinki Declaration, was approved by the Ethics Committee of Hamadan University of Medical Sciences.

The patients were instructed to brush their teeth and the teeth to be extracted were polished prior to extraction. Prep and drape and local anesthesia were performed. The patients were asked to rinse their mouth with 0.2% chlorhexidine for one minute immediately before extraction. After tooth extraction, the surgeon quickly separated the crown by a disk, while the assistant rinsed it with copious amounts of saline to avoid temperature increase and damage to the cells. The root was immediately transferred into a tube containing HBSS solution (Stem Cell Technology, Bio idea, Iran) without touching its outer surface. The tube was then immediately covered and taken to the laboratory.

To avoid contamination by gingival and pulpal cells near the coronal and apical portions of the periodontal ligament, PDL tissues attached to the middle third of the root surface were used. The method to culture stem cells was according to Gay et al.^17^ Periodontal ligament cells were scraped from the roots and placed onto a plate containing DMEM (Stem Cell Technology, Bio idea, Iran) and 15% FBS, and then enzymatically digested for 1 h at 37°C in a solution of 3 mg⁄mL of collagenase type I (Sigma, USA) and 4 mg/mL of dispase (Gibco, UK). The samples were then centrifuged at 400 g for 10 minutes and the plates were expanded in DMEM containing 15% FBS (Gibco, UK) and 1% penicillin/streptomycin (Gibco, UK) in six-well plates (NUNK, Denmark), followed by culturing at 37°C and 5% CO_2_ atmosphere. On day seven, adherent cells, which were 70% confluent, were washed twice with phosphate-buffered saline (PBS) and released from the culture surface using 0.25% trypsin-EDTA solution (Gibco, UK) and plated in tissue culture polystyrene flasks (Falcon, UK) at 5×10^3^cells/cm^2^. The primary cultures of both types of teeth PDLSCs mainly consisted of colonies of bipolar fibroblastoid cells. After the cells reached a clonal density of about 80‒90% (approximately 6‒8 days after primary culture) in order to double the culture system and to allow purification of mesenchymal cells, they were passaged. Cells in passages 2 to 4 were used, and the experiments were performed simultaneously in both groups.

### Evaluation of Colony Formation

Single cell suspension of PDL was seeded into six-well culture plates at 3.0×10^4^ cells/well in a colonogenic growth medium. The cultures were set up in triplicate and incubated at 37°C in 5% CO_2_ and >90% humidity for 12 days. For enumeration, day 12 colonies were washed twice with PBS and then fixed for 20 minutes in 1% (w/v) paraformaldehyde in PBS. The fixed cultures were then stained with 0.1% (w/v) toluidine blue for one hour and then rinsed with water and allowed to dry. Using a dissecting light microscope, aggregates of greater than 50 cells were scored as colony-forming unit fibroblasts (CFU-F) and counted.

### Cell Viability Assays

To determine viability of both cell populations (DePDL and PePDL), cells were seeded at a density of 2×10^4^ cells in 35-mm tissue culture dishes, cultured in standard media, and incubated (Binder, Germany) for 24 hours at 37°C in an atmosphere containing 5% CO_2_ for cell attachment and spreading. The MTT (3-(4,5-Dimethylthiazol-2-Yl)-2,5-Diphenyltetrazolium Bromide) method was used for this purpose. MTT assay is based on metabolic activity of cells. In this method yellow terazolium converts into purple insoluble formazon crystals by MTT succinate reductase system of mitochondrial respiratory chain that is active only in living cells. For MTT assay, the supernatant was replaced with 60 μL of MTT solution (mg/mL). Formazan crystals formed after 1‒1.5 hours of incubation at 37°C and 5% CO_2_. After solving the crystals in 300 μL DMSO the absorbance rate was measured at 540‒630 wavelength with an Elisa Reader.

### Flow cytometric analysis of cells

In order to identify mesenchymal stem cells in this study the flowcytometric (FCM) method was used for investigating the cell surface antigens CD105, CD90 and CD45.

After the third cell passage the cells were detached from the base of the plates by adding trypsin. The cell suspension was counted and 10^5^‒10^6^ cells were added to each vial. The vials were then filled with 1 mL of 3% PBS-BSA (Gibco Invitrogen Phosphate-buffered solution PBS, Sigma, Bovine Saline Albomine, BSA, UK, USA) solution. The suspension was centrifuged at 2000 rpm for 5 minutes. For each marker a test and control isotype vial was used. Antibodies (ABCAM, UK) were added accordingly; CD45 with a 1‒200 concentration was incubated (Binder, Germany) for 30 minutes at room temperature, and CD105 and CD90 markers with a 1‒50 concentration were incubated for 45 minutes at 37°C. A rabbit control isotype was incubated for 30 minutes at a 1‒200 concentration at room temperature. Afterwards PBS was added to each sample in order to reach a 1-mL volume. The suspensions were then centrifuged at 2000 rpm for 5 minutes (Hettich, Germany). The secondary antibody was added with a 1‒4 concentration and incubated for 45 minutes at 37°C. A 1-mL volume was then reached by adding PBS and centrifuged once more at 2000 rpm for 5 minutes. The cells were washed again with PBS and centrifuged. In the last stage the cell sedimentation was turned into a suspension with a 4% paraformaldehyde and stored at 2‒8°C until it was read under the flow cytometer (Beckton, Dickinson, USA).

### Osteogenic differentiation

After the third passage, cells in the developing adherent layer were used for osteogenic differentiation. When cell concentration reached 80%, they were used for differentiation by placement in a standard osteogenic medium (Stem Cell Technology, Bio Idea, Iran). During this period alkaline phosphatase activity test and alizarin red staining were performed on days 14 and 21, respectively.

### Alizarin Red Staining (ARS)

Alizarin red is used in a biochemical assay to determine, quantitatively by colorimetry, the presence of calcific deposition by cells of an osteogenic lineage. Mineralization of the cell layer was examined through the use of alizarin red staining method. On day 21 the cells were rinsed twice in PBS and fixed by covering with 10% formaldehyde and incubated at room temperature for 15 minutes. After rinsing in distilled water, the plates were stained with 1 mL/well alizarin red staining (Millipore, USA) solution, incubated at room temperature for at least 20 minutes, and rinsed in deionized water and 1‒1.5 mL of water was added to each well. Cells containing mineral deposits were stained with alizarin red solution and observed under an inverted microscope.

### Quantitative analysis of alizarin red staining

This analysis was performed on day 21 of subculture in osteogenic differentiation medium. According to the manufacturer’s recommendations, 400 μL of acetic acid was added to each well and incubated for 30 minutes while shaking. With the aid of a cell scraper, the cells were gently scraped from the plate and the cells and acetic acid were transferred to a 1.5 mL micro-centrifuge tube and then heated to 85°C for 10 minutes. The tube was transferred to ice for 5 minutes. The slurry was then centrifuged at 18,000 g for 15 minutes. The 10X ARS Dilution buffer was diluted 1:10 in distilled H_2_O and the 40 mM alizarin red solution was diluted 1:20 in 1X ARS dilution buffer. This yielded a 2 mM working stock. Standards could be constructed in a ‘high range’ or ‘low range’ set. Constructing the ‘high range’ set was carried out by diluting the 2 mM working stock in 2-fold serial dilutions in 1.5 mL micro-centrifuge tubes. To generate a ‘low range’ set, we began by first diluting the 2 mM working stock at 1:66 (15 μL of 2 mM alizarin red solution + 985 μL of 1X ARS dilution buffer) to achieve a 30 μM working stock. Construction of the ‘low range’ set was carried out by further diluting this 30 μM working stock in 2-fold serial dilutions in 1.5-mL micro-centrifuge tubes. The blank consisted of just the 1X ARS dilution buffer. When centrifugation ended, 400 μL of the supernatant were removed and transferred to a new 1.5-mL micro-centrifuge tube. The pH was neutralized and 150 μL of the standard/sample was added to an opaque-walled, transparent-bottom 96-well plate. It was read at OD=405 and values were recorded; each group was tested three times, and the mean value of the group was reported.

### Alkaline phosphatase activity

Cellular alkaline phosphatase (ALP) activity was assayed by colorimetric assay of enzyme activity with the substrate p-nitrophenolphosphate, according to Quarles et al^32^ on day 14th. In order to detach cells, they were washed twice with PBS and treated with 0.25% trypsin. Cellular ALP activity was determined using p-nitrophenylphosphate (90 mmol/L) as the substrate (pH=10.3) at 37°C for 30 minutes. The optical density value was then read at 405 nm.

For statistical analysis of our findings, Kolmogorov-Smirnov test was performed on the data, which indicated normal distribution of data in this study. Independent-sample t-test was used for comparing colony formation, viability (MTT results) and alizarin red staining results. In order to evaluate the effects of day and group on MTT, two-way ANOVA was used. The results for the alkaline phosphatase activity in the two main groups of this study and their controls in alkaline phosphatase activity test were comparatively evaluated using one-way ANOVA and post hoc Tukey tests.

SPSS 13 (Chicago, IL, USA) was used for data analysis and 0.05 was considered as the level of significance.

## Results

### Morphologic appearance, colony formation and viability

**Figure 1:**
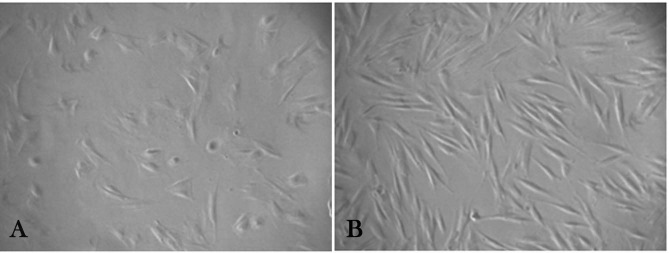
Morphologic appearance of primary cultures. (a): Permanent periodontal ligament stem cells; (b): Deciduous periodontal ligament stem cells under inverted light microscope. (×100 magnification) fibroblast-like populations were evident during in vitro expansion.

Pluripotent stem cell populations were successfully isolated from human PDL. These cells showed a mesenchymal type stem cell appearance; adherent spindle-shaped and fibroblast-like cells were seen at the bottom of the plates (Figure 1). SC populations isolated from both permanent and deciduous teeth were capable of forming adherent colonies.

A mean number of 36.5 colonies for the PePDLSC and a mean number of 35.5 colonies for the DePDLSC group with no significant differences were observed (P=0.485).

### Cell viability assay

According to MTT viability test results, DePDLSC cells showed the highest rate of viability on the third day but on day seven this rate was lower than PePDLSC, which was statistically significant (P=0.032) (Table 1). Overall comparison analysis of variance with two-way ANOVA showed differences between groups on different days (P<0.0001) but independent of time (day) ANOVA showed no significant difference between the two groups in this study (P=0.255).

**Table 1:** Comparison of mean MTT levels in permanent and deciduous PDLSC groups on days 3 and 7 using independent t-test

**Groups**	**Day**	**Mean**	**SD**	**P-value**
**Permanent**	3 3	0.2993	0.03155	0.000
**Deciduous**		0.3228	0.02742	
**Permanent**	7	0.7176	0.08147	0.032
**Deciduous**	7	0.6241	0.12179	

### Flow cytometry results

**Figure 2:**
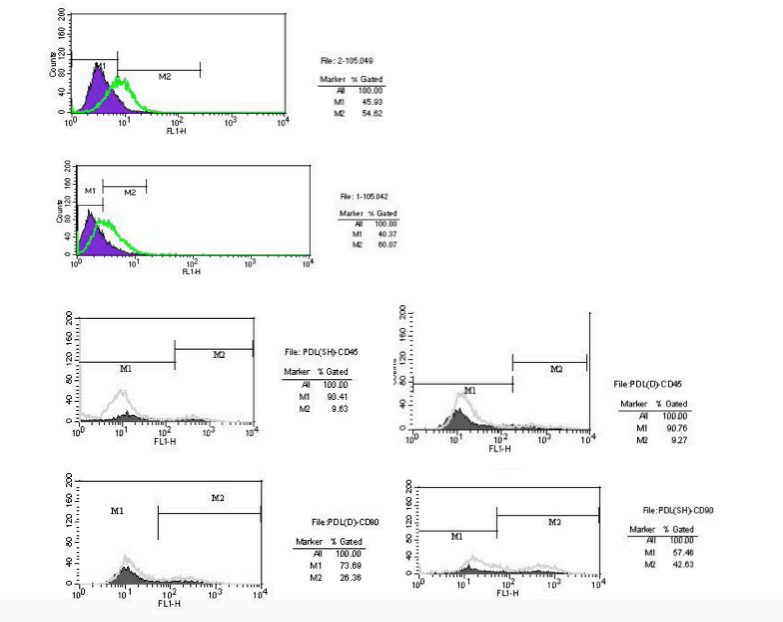
Flow cytometric test results. Stem cells from both deciduous and permanent periodontal ligaments are positive for mesenchymal stem cell markers of CD105 and CD90 but do not express the hematopoetic marker CD45.

In the flow cytometry analysis, the results of both groups of stem cells were positive for CD105, CD90 and negative for CD45 surface antigens (Figure 2).

### Mineral nodule formation

**Figure 3:**
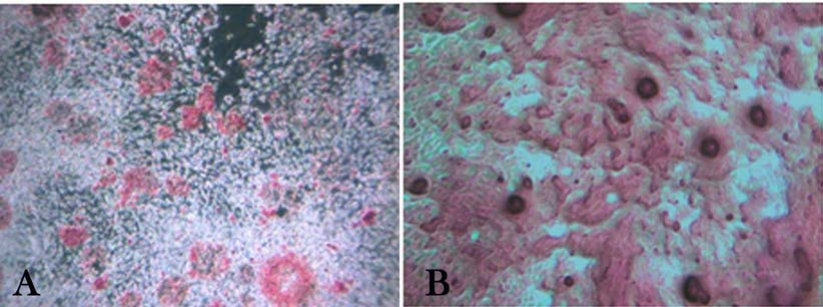
Osteogenic differentiation of deciduous and permanent periodontal ligament stem cells (stained with alizarin red on day 21 of culture). Bone nodule-like structures under an inverted light microscope, indicating calcium deposition in both groups of cell cultures; a) Deciduous periodontal ligament stem cell (×100); b) Permanent periodontal ligament stem cells (×200 magnification).

The two groups were differentiated into osteoblasts using osteogenic-induced medium; live cells organized in bone nodule-like structures were present and stained with alizarin red to determine calcium deposition (Figure 3).

### Quantitative analysis of Alizarin Red Staining

Alizarin red staining on day 21 revealed calcified deposits. A mean absorbance of 1677.83±148.62 for the permanent PDLSC samples and a mean of 1624.16±158.98 for the deciduous PDLSC were reported. No statistically significant difference was found between the two groups (P=0.559).

### Alkaline phosphatase activity test

This was measured after day 14 of subculture in osteogenic medium in order to quantify osteoblast differentiation. The results of one-way ANOVA in relation to the comparison between the two stem cell groups and their control samples indicated a statistically significant difference between the samples, in which permanent samples had higher alkaline phosphatase activity with a mean of 110.37 (m/MOL) compared to deciduous samples with a mean of 74.37 (m/MOL) (P<0.0001). Significant differences were also observed when these groups were compared with post hoc Tukey tests in which all the groups were significant except for the two control groups of each deciduous and permanent stem cell cultures (P=0.978).

## Discussion

In this study we comparatively evaluated some of the in vitro properties of mesenchymal stem cells derived from deciduous and permanent PDL tissues to find out whether they possess different in vitro properties that might influence their future in vivo applications.

Our flow cytometric analysis results showed that both groups of stem cells were positive for the CD105 mesenchymal marker, consistent with previous investigations.^4,28^ The proportion of CD105^+^ cells in PePDLSC was 60.07%, and 54.62%. For the DePDLSC samples, this proportion has also been reported to be higher in DePDLSC in previous reports. But the differences in the percentages reported in the studies with ours could be due to the different flow cytometric devices utilized and sample preparation.^28,29^

The samples were both positive for CD90 and negative for CD45 surface markers, indicating they were mesenchymal stem cells.

Both isolated cell populations were able to form colonies and differentiate into osteoblasts within in vitro osteogenic medium. This has also been reported in previous studies on permanent PDL stem cells by Nagotomo et al^33^ and Gay et al,^17^ and reports of deciduous stem cell isolations by Silverio et al and Song et al and Ji et al.^28,29,30^

Regarding the in vitro capability of colony formation and viability comparison between these two types of stem cells, the DePDLSC showed higher early viability of 0.3228 on day three compared to 0.2993 (P<0.000) but this was not significant on day 7 (P=0.032). Overall there was no statistically significant difference between the groups regarding viability of stem cell markers and colony formation. This is comparable to a recent study by Song et al, in which they also reported no significant differences between the proliferation rate, cell cycle distribution and expression of stem cell markers such as Stro-1 and CD146 in PePDLSC and DePDLSC in vitro.

The higher early viability of the DePDLSC observed in our study is comparable to some extent to the results achieved by Silverio et al, who reported DePDL-CD105^+^ subsets to be more proliferative compared to PePDL subsets. They found that after 10 days in culture, the cell number of DePDL populations was almost 3 times higher compared to the PePDL populations, which could be attributed to the younger donor age in deciduous teeth, considered to be a factor affecting stem cell properties in previous studies.^23,24^

Interestingly, comparison of differentiation potentials by Song et al^27^ showed that the PePDLSC transplants produced more typical cementum/PDL-like tissues and expressed more cementum/PDL-related genes compared to DePDLSCs, suggesting that PePDLSCs are better candidates for use in reconstructing the periodontium. In our study we were also able to find a higher alkaline phosphatase activity of PePDLSC compared to DePDLSC. Previously Chadipiralla et al^36^ also reported higher calcium deposition of PDLSC compared to SC derived from pulp of deciduous teeth (SHED) after Retinoic acid-induced osteogenic differentiation. However, in this study the alizarian red quantitative analysis revealed no statistically significant differences between the permanent and deciduous cells although the amounts were slightly higher in permanent PDL stem cells. This interesting finding could be due to the nature of these teeth, which are to be in function for longer periods in which they should be able to regenerate and preserve the periodontium. Interestingly, a recent study by Li et al showed induction of osteoclast activation and root resorption by deciduous-derived stem cells.^37^

As previously mentioned, our results showed no significant differences in the amount of colony formation between the two groups (P=0.485). This is similar to the recent report of Song et al^29^ but different from that reported by two other studies.^28,34^ Some studies have found an age-related decrease in osteoblastic, but not adipogenic, differentiation in human bone marrow-derived mesenchymal stem cells.^38,39^ Moreover, it seems that bone marrow-derived mesenchymal stem cells from younger donors demonstrate an increasing proliferative rate and adipogenic differentiation capacity.^40^ A higher expression of adipogenic-related genes was also observed in DePDL cells in Silverio’s study, whereas PePDL-CD105^+^ cell subset exhibited a more homogeneous osteoblast/cementoblast response.

Due to our limitations we were not able to investigate in vivo properties comprehensively and compare clinical application potentials in this study. Although previous studies and our results suggest PePDSC to be better candidates for periodontal regeneration, we suggest further in vivo studies to also determine the real impact of type of dentition and differences in immunological properties of stem cells derived from PDL on their properties and differentiation potentials.

## Conclusion

Mesenchymal stem cells were successfully isolated from the PDL of deciduous and permanent teeth. No statistical differences were found in stem cell markers, colony formation and viability. Our findings indicated a minor in vitro difference in osteogenic differentiation properties of PePDLSC, which was slightly higher than DePDLSC and this might be considered as a factor affecting their future clinical applications.

## Acknowledgments

None

## Authors’ contributions

MK contributed to developing the original idea, the study design and sample collection. IA preformed all the laboratory cell culture and differentiation procedures. LG contributed to the development of the study design and protocol, sample collection, data collection and analysis and manuscript preparation. MK and IA critically revised the manuscript for intellectual content. All the authors have read and approved the final manuscript.

## Funding

This study was a research project funded by Hamadan University of Medical Sciences.

## Competing interests

The authors declare no competing interests with regards to the authorship and/or publication of this article.

## Ethics approval

This study was approved by Hamadan University of Medical Sciences Research Committees and Ethics Board (IR.UMSHA.REC.91.0129494).
